# Network Effects of Demographic Transition

**DOI:** 10.1038/s41598-019-39025-4

**Published:** 2019-02-20

**Authors:** Tamas David-Barrett

**Affiliations:** 1 Universidad del Desarrollo, Facultad de Gobierno, CICS, Av. Plaza 680, San Carlos de Apoquindo, Las Condes, Santiago de Chile 7610658 Chile; 2 University of Oxford, South Parks Road, Oxford, OX1 3UD United Kingdom; 3 Kiel Institute for the World Economy, Kiellinie 66, D-24105 Kiel, Germany; 4 Population Research Institute, Väestöliitto, Kalevankatu 16, Helsinki, 00101 Finland

## Abstract

Traditional human societies use two of biology’s solutions to reduce free-riding: by collaborating with relatives, they rely on the mechanism of kin-selection, and by forming highly clustered social kin-networks, they can efficiently use reputation dynamics. Both of these solutions assume the presence of relatives. This paper shows how social networks change during demographic transition. With falling fertility, there are fewer children that could be relatives to one another. As the missing kin are replaced by non-kin friends, local clustering in the social network drops. This effect is compounded by increasing population size, characteristic of demographic transition. The paper also shows that the speed at which reputation spreads in the network slows down due to both falling fertility and increasing group size. Thus, demographic transition weakens both mechanisms for eliminating free-riders: there are fewer relatives around, and reputation spreads slower. This new link between falling fertility and the altered structure of the social network offers novel interpretations of the origins of legal institutions, the Small World phenomenon, the social impact of urbanisation, and the birds-of-a-feather friendship choice heuristic.

## Introduction

The ability and propensity to make friends, that is, lasting, non-kin, positive social affiliations, is a human universal^1–5^, with deep evolutionary roots^6–9^. Despite this, humans, like most animals^10^, prefer collective action with kin^11–16^, similar to elephants^17^, African wild dogs^18^, and bottle nose dolphins^19^. People prefer the majority of their social contacts to be kin rather than friends whenever it is possible^20–23^. For instance, in contemporary forager cultures (such as the Ache^24^, Ju/’hoansi^24^, Agta^25^, and Mbendjele^25^), the average member of an average band is connected to about three-quarters of the other band members either via direct biological or affinal, through-marriage, link^24^. This ratio increases further in traditional agriculturalist societies^25^. Most of the social world of traditional societies in general is dominated by direct or affinal kin, especially when it comes to organising collective action^26–29^.

Almost all modern societies have gone through demographic transition during the past two hundred years^30–32^, characterised by falling mortality followed by falling fertility, resulting in increased population size and permanently low fertility^30,32–34^. A trivial social consequence of decreasing fertility is that it reduces the number of relatives. For instance, if the average fertility per woman is 5, then an average individual will have 4 siblings, and 40 first-degree cousins (5 offsprings on average for each of the four siblings of the mother and the four siblings of the father). If, however, fertility falls to 2 per woman then the average individual will have 1 sibling, and only 4 cousins. Thus, a shift from total fertility rate (TFR) of 5 to TFR of 2, the typical start and end points during demographic transition, reduces the number of same-generation relatives with whom a grandmother is shared from 44 to 5. As the total number of relatives is dependent on the population’s fertility, the available set of kin is dramatically reduced during demographic transition. Even if people would prefer to include kin more than non-kin in their social network as much after the transition as before, in low-fertility societies there are just not enough kin to serve as social contacts^35^.

Although the effect of falling fertility on many aspects of the society has been much discussed^36,37^, the literature about the relationship between social networks and fertility has been in one causal direction so far: the focus has been on the way social networks mitigate attitudes towards child bearing^38–41^. In fact, part of the much-contested literature^34^ concerning the origins and mechanism of demographic transition suggested that falling fertility is a network phenomenon^42,43^, supported at least in part by culture-specific empirical evidence^44,45^. The empirical literature has established that a fall in the number of children can impact the structure of the social networks by showing that kin contacts decline with demographic transition and industrialisation^46–48^. If and how replacing kin with friends in societies characterised by falling fertility affects the structure of social networks is a question that has been so far unexplored.

Given that independent of culture, humans tend to prefer ego group sizes with relatively small individual variation^49–52^, people living in low fertility societies find themselves in a deficit of social contacts when the number of possible relatives falls with demographic transition^35^. This problem is solved by replacing the missing kin with non-kin friends. This is helped by the fact that the method of bonding, that is, making and maintaining friends, is via frequent meaningful meetings and interactions, similar to maintaining relationships among biological relatives^53,54^.

**Figure 1:**
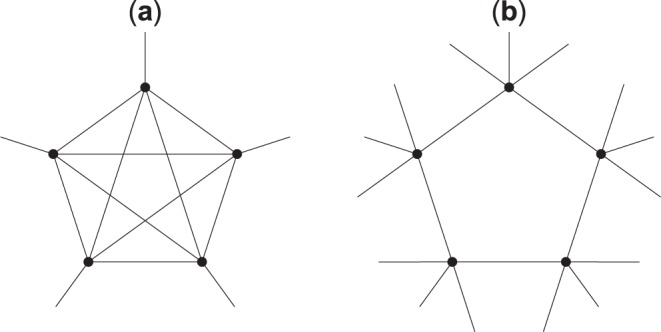
Illustration for how the clustering coefficient can change even when the degree, i.e., the number of connections, is unchanged. Panel (a) is a stylised representation of a high-fertility society in which five agents are depicted, who are all connected to one another and, in addition, to one further person each. Panel (b) also depicts five agents; these are, however, only connected to two other agents within this circle, and an additional three others outside the circle and not connected to each other. This panel is the stylised representation of a low-fertility and/or urbanised society. Note that the network degree of each agent is five on both panels, i.e., each agent in the circle has five social connections. Yet, the clustering coefficients of all agents, i.e., the number of closed triangles out of all the possible triangles in their individual social network, is 50% in Panel (a) and 0% in Panel (b).

Having non-kin friends solves the problem of how to achieve the desired number of social contacts, but raises a completely different issue. As a simple thought experiment shows, the structure of the social network around an individual who is surrounded by relatives is likely to be different from one surrounded by friends. The reason is that an ego’s relatives are more likely to be relatives to one another than the ego’s friends are to be friends themselves. For instance, if an individual has two full siblings, i.e., sharing both parents, then, by definition, they themselves will be siblings to each other. However, if an individual has two good friends, although it is possible that these friends will be friends with each other, this is far from certain. This phenomenon is likely to have an impact on the structure of the social network people live in (Fig. 1).

This paper investigates how the social network structure changes when individuals replace kin with friends as a consequence of falling fertility.

## Network Structure – Methods

Let us create a population of many generations, in which the biological relatedness is tracked. For this, each individual is defined by the following record:

1 $$\{i,g,{F}_{i}\}$$

where *i* is the index number of the agent, ${g}_{i}\in \{0,1\}$ is the gender of the agent, ${F}_{i}=\{{p}_{i,1},{p}_{i,2},{p}_{i,3},{p}_{i,4},{m}_{i},{f}_{i}\}$ is the set of the index numbers of the agent’s four grandparents, mother and father.

Let *I*_*s*_ denote the set of agents belonging to generation *s*. Let us assume that the agents form heterosexual pairs among unrelated individuals:

2 $${P}_{s}=\{\{a,b\}|{g}_{a}\ne {g}_{b}\,{\rm{and}}\,a,b\in {I}_{s}\,{\rm{and}}\,{F}_{a}\cap {F}_{b}=\varnothing \}$$

where *P*_*s*_ is thus the set of pairs formed in generation *s*. (Notice that the above pair-formation rule implies that if the number of females and males is not even, some of the more numerous gender do not find a partner. The model also assumes that the pairings last a life-time, and are monogamous).

Let *k* denote the fertility of any single pair, and assume that it is a Poisson-distributed random variable:

3 $${k}_{{p}_{s}}\sim \mathrm{Pois}(\kappa )$$

where *κ* denotes the expected value of fertility, and thus the average fertility in the population.

Let *B*_*x*,*y*_ denote the set of children born to the pair formed by *x* and *y*:

4 $${B}_{x,y}=\{\{j,g\{{m}_{x},{f}_{x},{m}_{y},{x}_{y},x,y\}\},g\sim {\rm{U}}\{0,1\}\}$$

where the gender of a child is random, uniformly distributed.

Let *B*_*s*_ denote all the births to a generation:

5 $${B}_{s}=\{{B}_{x,y}\}{|}_{\{x,y\}\in {P}_{s}}$$

Notice that the expected size of this set is as follows:

6 $${\rm{E}}[\#{B}_{s}]\approx \frac{\kappa {n}_{s}}{2}$$

where *n*_*s*_ denotes the population size in generation *s*, and E is the expectations operator. (The expected size is only approximate, as a small fraction of the population does not pair up due to the random number of the two sexes, and the assumption that the pairings are monogamous).

Thus, if the fertility of the population is larger than replacement, the size of the population increases generation by generation.

Using equations (1) to (6) and starting with an initial population of 50–100, I simulated, using Wolfram Mathematica, the population dynamics in successive generations, varying the fertility, *κ*, between 2.5 and 5.0. (The upper limit of this range is given by the average fertility in traditional hunter-gatherer societies^55^, while the lower end reflects a computational reason: at 2.5 the populations do not collapse during simulation).

I ran each simulation until the total population size exceeded a target number (500 or 2000). In most cases the final generation’s population size exceeded the target number. When this was the case, I eliminated randomly chosen agents so that the final population size was exactly in line with the target number. This way I had built a library of simulated populations, each being a list of agents’ records containing the index numbers of their parents and grandparents as defined in equation (4).

Next, I built a binominal adjacency matrix, *a*, for each population in the library, assuming that the agents regard each others as kin if they share at least one grandparent:

7 $${a}_{i,j}=\{\begin{array}{c}1\,{\rm{if}}\,{{\rm{F}}}_{i}\cap {F}_{j}\ne \varnothing \\ 0\,{\rm{if}}\,{{\rm{F}}}_{i}\cap {F}_{j}=\varnothing \end{array}$$

Notice that in most hunter-gatherer societies, relatives are recognised and tracked on average up to second cousin^56^. For computational reasons, I assumed kin recognition up to first cousin only. (Otherwise the target group sizes would have had to be unrealistically large: and would take too long to compute all the steps). There is no reason why the theoretical observations of this paper would be different with the cut-off being at first-degree as opposed to second-degree cousins.

Trivially, the adjacency matrix *a* is diagonally symmetric, for if *i* is related to *j*, then by definition *j* is also related to *i*:

8 $${a}_{i,j}={a}_{j,i}\forall i,j=1,\ldots ,n$$

Thus the adjacency matrix, *a*, defines a graph in which each edge is undirected, and corresponds to a mutually recognised kin relationship:

9 $$g=\{i\leftrightarrow j\}|i,j=1,\ldots ,n\,{\rm{s}}{\rm{.t}}.{a}_{i,j}=1$$

Let *d*(*g*) denote the average degree of the graph *g*:

10 $$d(g)=\frac{\sum _{i=1}^{n}\sum _{j=1}^{n}{a}_{i,j}}{2n}$$

Notice that the average number of relatives is dependent on the fertility parameter, *κ*. If there are more children born, there are more relatives.

(See Fig. S1a for the relationship between *d(g)* and *κ*).

Let us assume that each agent has a uniform need for and capacity to maintain a certain number of social contacts. This assumption is in line with empirical evidence suggesting that, due to the limited social time budget^57^, the number of meaningful social contacts of an average human lies within a relatively narrow range^58–60^, and is a standard assumption of the behavioural synchrony model family^61–63^. Let *ν* denote this parameter. I assume that this social capacity parameter is set arbitrarily at *ν* = 60. (I chose this level as it is just a little higher than the average number of relatives in the upper end of the fertility range in the simulations. Ethnographic demography suggests that in hunter-gatherer societies most social contacts are recognised as either close or distant biological kin, or affinal kin through marriage^24–29^, and there is only a relatively small space for non-kin friends. This suggests that a limit just above the number of recognised kin in the highest fertility case is, albeit arbitrary, qualitatively correct). For agents that had a higher number of relatives than the limit, *ν*, I eliminated a random set of relatives up to the limit. (See Fig. S1b for the relationship between *d(g)* and *κ* after this truncation).

Because *ν* is chosen so that the number of recognised relatives is just under this limit in the case of the high-fertility population, depending on the simulation fertility parameter, *κ*, the agents are almost always short of social contacts, to a varying extent. To fill the gap, the agents were assigned random “friends”. To do this, I added random edges between unrelated agents to the adjacency matrix (and as a consequence, to the social network graph), until each agent’s number of social contacts was at the capacity, *ν*.

Let *b* denote the adjacency matrix of all connections between social contacts, and *h* the corresponding social network graph. Due to the graph-generation method, the degree of each agent in the *h* graph is uniform:

11 $$d(h)\equiv v$$

In this way I created a library of pairs of *a* and *b* adjacency matrices and corresponding pairs of *g* and *h* social network graphs. Notice that because *g* is the network of relatives, and *h* is the entire social network which includes all relatives, *g* is a subgraph of *h*.

Let *δ*_*i*,*j*_ denote the shortest path between nodes *i* and *j* in the graph *h*. Let *χ*_*i*_ denote the local clustering coefficient of agent *i*:

12 $${\chi }_{i}=\frac{\#\{j,k\}|{\delta }_{j,k}={\delta }_{i,j}={\delta }_{i,k}=1}{nv(v-1)}$$

that is, the number of triadic closures around agent *i* in proportion to all possible such triads.

Using this definition, let *χ* denote the average local clustering coefficient of graph *h*:

13 $$\chi =\frac{1}{n}\sum _{i=1}^{n}{\chi }_{i}$$

And let *δ* denote the average graph distance among the vertices of graph *h*:

14 $$\delta =\frac{1}{n(n-1)}\sum _{i=1}^{n}\sum _{i\ne 1}{\delta }_{i,j}$$

## Network Structure – Results

**Figure 2:**
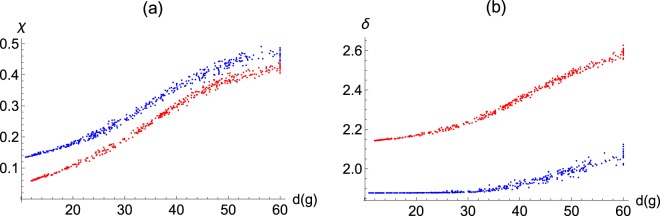
When friends replace relatives, local clustering falls, and the graph distances become shorter. In both panels, the x-axis is the average number of relatives an average individual has. (At the upper limit of 60, all the social contacts of the individuals are relatives). Panel (a) y-axis: the average local clustering coefficient, i.e., the average number of closed social triads around individuals. Panel (b) y-axis: the graph distance, i.e., the average length of shortest routes between nodes in the network. Blue: group size at 500. Red: group size at 2000. (Each colour on each panel represents 400 independently simulated repeats).

Falling fertility changes the structure of the social network in two ways: as the average degree of the kin-only graph, *d*(*g*) falls, both the average local clustering, *χ*, and the average graph distance, *δ*, decreases (Fig. 2). In other words, the lack of relatives results in fewer closed triads, i.e., social contacts are less likely to be linked to each other, but also there are fewer steps between unconnected agents.

Notice that group size plays an important role in both the fertility-clustering and the fertility-graph distance relationships. First, local clustering is lower in larger groups, due to the fact that friends are chosen from a larger population, and hence are less likely to be social contacts to one another (Fig. 2a). With falling fertility, local clustering decreases faster in larger groups (Fig. S2a). Second, while larger groups have higher graph distances (Fig. 2b), falling fertility decreases graph distances faster in larger groups (Fig. S2b).

**Figure 3:**
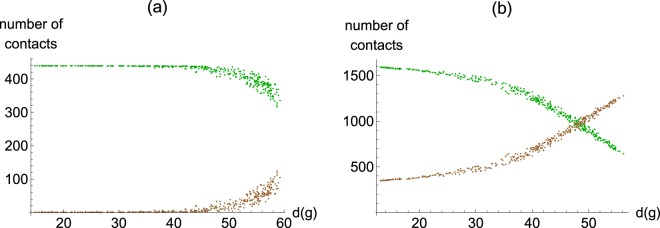
The number of contacts in two- and three-step distances. Panel (a) group size at 500; panel (b) group size at 2000. Green: the number of contacts of an average ego which are two steps away in the social network of the ego. Brown: the number of contacts three steps away. (Each colour on each graph represents 400 independently simulated repeats).

A different way to illustrate the changing graph distance phenomenon is via assessing how many others agents can reach in a given number of steps. Trivially, there is only one agent at a zero-step distance: the ego herself. At a one-step distance is her set of social connections, fixed at *ν* = 60 due to the assumption that the number of social contacts does not vary as a function of fertility. As fertility falls, the number of agents that are two steps away from the ego increases in both group sizes (Fig. 3). At the same time, those that are three steps away reduce in number. In other words, many agents that would be three steps away before falling fertility, become only two steps away. The set of friends of friends enlarges.

For instance, in the case of the 2000-sized groups (Fig. 3b), the number of two-step away agents triples from the highest fertility societies to the lowest fertility ones. At the same time, the number of three-step away agents falls to one-third of the original.

## Reputation Dynamics – Methods

During demographic transition, fertility falls and population size increases, which, as shown above, changes the structure of the social network. Although the network degree, i.e., the number of social partners of the agents, is unchanged, the local clustering coefficient decreases both as fertility falls and as group size increases. To see how this alteration in the network structure changes the speed at which social information spreads in the network, let us consider the following reputation regime.

Let the *n* × *n* size matrix *r* denote a reputation matrix, where *r*_*i,j*_ ∈ {0, 1} is agent *i*’s belief of agent *j*’s type. Let us assume that in round 0, all agents have a reputation of being type 0 in the eyes of all others:

15 $${r}_{i,j}^{0}=0\forall i,j=1,\ldots ,n$$

In round 1 a randomly selected agent, denoted by *xi*, is observed by one randomly selected social contact of his, denoted by *xj*, as type 1. (For instance, *xi* cheats on *xj* in a game and thus reveals himself to her as untrustworthy). Formally:

16 $${r}_{xi,xj}^{1}=1$$

where

17 $$xi\sim {\rm{U}}\{1,\ldots ,n\}$$

and

18 $$xj\sim {\rm{U}}\{j|{b}_{xi,j}=1\}$$

In each successive round, the agents “gossip” with their contacts about shared acquaintances (either friends or relatives). They update their beliefs such that, if either of them thinks that the acquaintance is a cheater then they both update their belief to cheater. Let *α* denote the probability that such gossip takes place among a pair of contacts.

Formally, for agent *i* let *β*_*i*_ denote the number of agents that are at the same time (i) connected to agent *i*, and (ii) connected to agent *xi*, and (iii) and have a reputation of *xi* as 1:

19 $${\beta }_{i}^{t}=\#\{j|{b}_{i,j}=1,{b}_{j,xi}=1,{r}_{j,xi}^{t}=1\}$$

Then let agent *i* update her belief about *xi* in the following way:

20 $${r}_{i,xi}^{t+1}=\{\begin{array}{c}{r}_{i,xi}^{t}\,{\rm{if}}\,{b}_{i,xi}=1\\ 1\,{\rm{at}}\,{\rm{prob}}\,{\rm{1}}-{(1-\alpha )}^{{\beta }_{i}^{t}}\,{\rm{and}}\,{r}_{i,xi}^{t}\,{\rm{otherwise}}\,{\rm{if}}\,{b}_{i,xi}=0\end{array}$$

Notice that in this reputation dynamics 0 can only turn to 1, not the other way around. In this version, there is no forgiving.

The interpretation of the reputation mechanics is that in a group of co-operators a defector emerges, whose true nature is observed by one contact. The question that this model answers is how fast and how far the news travels in the network. The role of *α* can be interpreted as either transmission probability, or the likelihood that an agent adopts the belief of a social contact. In the latter case, *β* can be interpreted as a propensity for the agent to follow the wisdom of the crowd.

## Reputation Dynamics – Results

**Figure 4:**
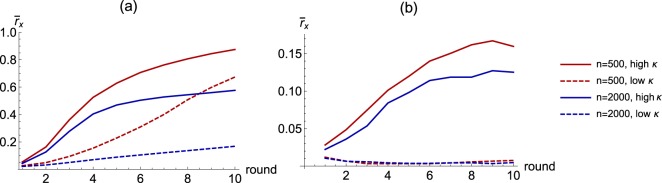
Reputation dynamics on mixed friend-kin social networks. Panel (a): reputation dynamics without forgiving. Panel (b): reputation dynamics with forgiving. Low fertility is defined as the number of relatives being less than one-third of total social contacts: *d*(*g*) < 1/3 *ν*. High fertility is defined as the number of relatives being higher than one-third of all social contacts: *d*(*g*) > 1/3 *ν*. (α = 0.1, π = 0.50. Number of graphs in the library: 400 for both n = 500 and n = 2000).

Both fertility and population size affect the speed and extent of reputational dispersion (Fig. 4a). First, independent of population size, reputation travels faster in high-fertility compared to low-fertility societies. Second, independent of fertility, reputation travels slower in larger populations.

Thus, if demographic transition is characterized by falling fertility and increasing population size, then the compound effect is a weakening of the reputation effect. If this reputation is about the stance to cooperate or free ride in a costly collective action (i.e., 0 for cooperative stance and 1 for free riding stance), and if the reputation effect is what maintains the cooperative stance, then both falling fertility and increasing population size should lead to an increase in free riding.

In the above reputation mechanics I assumed that once a reputation belief turns from 0 to 1, there is no change back. It is interesting to consider an alternative mechanism characterised by forgiving (or forgetting). Let us assume that in each round, after the updating of equations (19)-(20), each belief 1 can be turned, independently, into 0 at probability *π*.

21 $${\rm{\Pr }}[{r}_{i,k}^{t+1}=0]=\pi $$

Given that the forgiving possibility is independent among individual contacts, it is not surprising that forgiving is likely to take place only when a small number of agents have turned their beliefs to 1 (Fig. 4b). As a consequence, high-fertility populations never really forgive, while low-fertility populations forgive (or forget) fast.

This reinforces the qualitative results of the version of reputation mechanics that does not include forgetting: falling fertility during demographic transition is likely to undermine the reputation-based punishment of free riding.

## Discussion

During the past two centuries, most contemporary societies have gone through demographic transition^30–34^. Before the change, mortality was high, and so was fertility: many children were born, many died, and the two variables more or less balanced out. When the transition began, first mortality fell, which, usually a generation or more later was followed by a drop in fertility. As a consequence, by the end of the transition, the society had a larger population, while family size shrank.

The first model presented in this paper shows that these two outcomes of demographic transition, i.e., a large population made up of small families, restructures the social network in two key ways. First, both the increased population size, and the decreased fertility result in a drastic fall of the local clustering coefficient. In traditional societies, characterised by high fertility and small group size, the majority of social contacts of an average individual are likely to be relatives and thus also likely to be connected to each other. With demographic transition, this changes: an individual’s social contacts are less likely to be social contacts with each other. Second, the falling clustering probability also results in decreasing graph distance among members of the society. After demographic transition, the number of others that can be reached in two steps increases sharply. These structural changes in the social network altered the world of the societies that underwent demographic transition.

By describing the ego-scale change in the texture of the social network resulting from fertility transition, the model spans out a framework in which some social phenomena that have not previously been regarded as related to demographic processes may be re-examined. In particular, it is possible that it was the falling graph distance that led to the rise of the Small World phenomenon; and that falling clustering weakened the reputation-based social norm enforcement typical of small-scale, high-fertility societies. I discuss these two phenomena below.

### Small World

In the decade between the mid-1990s and the mid-2000s, a new research field emerged: the statistical analysis of the social network among millions of people^64^, based on the rise of social networking sites and the emergence of network analysis as an academic discipline^65,66^. One key early finding was that the Small World phenomenon holds for large societies, and maybe the entire global population^67^.

This paper shows that the Small World phenomenon could have been caused by the pattern of falling fertility that first characterised Western Europe starting 200–300 years ago, which from the middle of the 20^th^ century became a global phenomenon. The global population’s average fertility fell from 5.2 children per woman to 2.5 children between 1960 and 2010^68^. A secondary effect, if any, comes from any method that allows an increase in social contacts, which social networking sites probably do, although the size of this effect is much debated^69–71^. In other words, the Small World phenomenon can be attributed to demographic transition rather than to internet-based social networking sites. The latter, together with the new science of social network analysis, merely allowed the recognition of the phenomenon.

### Social norm enforcement

All social species have a problem of organising collective action. Sometimes the solution is dependent only on the ability to coordinate movement, e.g., a flock of fish or a herd of ungulates balancing predator avoidance or moving between patches of resources. Many species, however, rely on the costly contribution of individuals, and thus face the problem of how to ensure that group members pull their weight. Unless some solution emerges to deter free-riding, collective action collapses. Two main solutions have been invented by evolution: inclusive fitness^72–74^ and reputation dynamics^75,76^. The former relies on the presence of biological relatives, while the latter requires a stable social network with high local clustering. Traditional small-scale, high-fertility societies use both of these tricks when they organise collective action via kinship networks^35^. This paper shows that falling fertility in demographic transition affects both pathways: fewer children mean that families are smaller, and thus the individuals’ social networks are necessarily filled with friends more than with family; and at the same time, falling local clustering slows the spread of social information within the network.

In other words, demographic transition weakens the traditional methods of norm enforcement. One solution to this problem has been the emergence of the modern legal system, that is, an institutional framework that changes the incentives by altering individuals’ payoffs via standardised third-party punishers. In particular, the model’s result suggests that rules guiding interactions in collective action are likely to emerge in parallel with the shift from kin-based to non-kin-based social networks.

### Urbanisation and Law

This paper assumes that agents form social connections with others that they recognise as relatives before they fill any gap in social contact numbers with non-kin friends. This assumption is based on empirical literature showing that both non-human social animals as well as humans consistently prefer to form social connections with relatives over non-relatives^10,13,16–18,22,77^. However, although as a general approximation the assumption of strong preference of network edges for kin over non-kin friends is well founded, empirically the relationship is not absolute^24,25,53,54^. In fact, the frequency of meaningful interaction affects the emotional closeness with both kin and non-kin^54^, mediated at least in part by geographical distance^21^.

To recognise this effect, let the concept ‘effective fertility’ denote the level of virtual fertility that corresponds to the number of relatives that agents have in their actual social network. Note that in a traditional society, the effective fertility is the same as actual fertility. However, in non-traditional societies it may not be so. If, for instance, others than relatives live in between an individual and her kin, frequent meaningful interaction will facilitate the formation of social network ties with these non-kin. Thus, real relatives are crowded out by spatially in-between non-kin.

This phenomenon is likely to occur in urban spaces, as well as in displaced, migrant populations. In all of these examples, the individuals lose connections to their real relatives even if they exist. For instance, in an urban space, maintaining meaningful relationships with relatively distant kin is likely to be more costly. Similarly, displaced or migrant populations are likely to have left at least some of their kin behind. A similar impact can result from epidemics if they affect the population evenly and, following the same logic, also from warfare. As a consequence, the available number of kin with whom the individuals could populate their social networks, falls. The effect on the social network is equivalent to real fertility falling.

Thus, urbanisation triggers a change in the social network structure, similar to decreasing fertility, without an actual drop in the number of children born. In an urban space, effective fertility falls without actual fertility changing. If this is true, then with urbanisation the average graph distance in the social network decreases, creating a Small World effect, while the social network’s local clustering falls, weakening the social sanction for norm-breaking, free-riding. This may explain why ideas travel faster in cities^78^, innovation rates correlate with city density^79^, and that the first codification of laws has been associated with the rise of early cities^80^. Furthermore, the falling clustering coefficient might explain some of the social factors of why people living in cities are more likely to feel lonely, and suffer from depression^81–83^.

### Birds of a feather

The second key assumption of the model is that while the agents’ relatives are determined by biological lineage, the friends who fill the gap in social connections are chosen at random. Indeed, people tend to be born into kin networks, and thus, the assumption that the agents’ set of relatives is pre-determined is plausible. However, in reality, friends tend not to be chosen as a random draw from the non-kin pool of the population. Humans prefer friends with whom they share cultural heritage, preferences, tastes^20,84,85^.

If at least some of these characteristics are passed on within families from one generation to the next, then non-kin friends chosen for their similarity are likely to be less distant in the social network than a person drawn entirely randomly. To the extent this is the case, associative friendship choice counteracts the structural effect of falling fertility on the social network. However, as long as there is at least some level of randomness in kin-replacing friendship formation, the birds of a feather effect merely moderates rather than eliminates the phenomena predicted in this paper.

As higher local clustering results in an increased incentive not to free-ride on each other, and thus strengthens the network reputation effect, it is the interest of individual agents to increase the local clustering around them. In fact, this is exactly what people tend to do when they act in a way that results in triadic closures around them^86^. “I have somebody you must meet. You two will hit it off mightily” is a line that sounds as if making the new acquaintance was the interest of the two friends previously unknown to each other. That may be; however, if triadic closure is achieved in this way, it might benefit the introducer more than those introduced. For, if the new social tie becomes active, the introducer’s local clustering coefficient is guaranteed to increase, while that of the two new acquaintances may not (if the price of the new friend is dropping an old one in a way that this breaks up a triad).

Humans regularly engage in social activities that are likely to increase the local clustering in their social network. It may be having a hobby, joining a club, playing a team sport^87–89^. In fact, maybe the ultimate motivation for choosing a friend that is similar in some key characteristics is that this results in triadic closure^90,91^.

It is also possible that people adapt to the falling clustering coefficient by focusing on a select few social contacts. This friendship formation heuristic goes to the core of two important further assumptions of the paper: (i) that the number of social contacts is fixed, and (i) that all social contact links are unweighted, i.e., an edge either exists or it does not, and thus they are homogenous across the graph. This is not necessarily the case.

Recent empirical evidence suggests that as social networking sites and apps reduced the cost of maintaining social relationships, the number of social contacts, of any form, has increased considerably^92^. If the new social contacts are loosely linked friends, the first model of this paper suggests that social networking sites would decrease the clustering coefficient, albeit not the number of closed triads.

At the same time, empirical evidence suggests that the number of social contacts with whom the connection is strong, i.e., the core social network of individuals, has decreased in size both in offline networks of increasingly urban populations^93^, as well as on social networking sites^94^. This paper’s results suggest that such an increasing focus on the close network could be hypothesized as an adaptive response to falling fertility and increasing rate of urbanisation. The modelling consequences of this dual process are for future work.

In summary, the models of this paper introduce a new, causal link from demographic processes such as fertility transition, urbanisation, epidemics, warfare, and migration to social trust, and the emergence of legal institutions. The shared characteristic of all these demographic phenomena is that kin are replaced by friends either because fewer relatives were born as in the case of falling fertility, or because the relatives that are alive are not available as in the case of urbanisation and population displacement, or they have died as in the case of epidemics and warfare. As the missing kin are replaced by friends, the social network’s clustering coefficient falls, weakening the traditional mechanisms for eliminating free-riders. It is this mechanism that links these phenomena to falling societal trust, social alienation, and ultimately, the rise of a new way of ensuring collective action among non-kin: institutionalised norm enforcement. Thus, it is demographic processes that led to the weakening inclusive fitness-based, and network reputation-based solutions to the costly collective action problem, resulting in the emergence of a third solution: legal institutions.

## Supplementary information

Supplementary Information

## Data Availability

This is a theoretical paper, there is no data associated with this manuscript.
