# Supplementary material for: Network Effects of Demographic Transition
Source: Sci Rep. 2019 Feb 20;9:2361. doi: 10.1038/s41598-019-39025-4 (PMC6382786; doi:10.1038/s41598-019-39025-4)
Supplement: Supplementary file 1 — Supplementary Information [file 41598_2019_39025_MOESM1_ESM.pdf]

Supplementary Information for Network Effects of Demographic Transition

Tamas David-Barrett

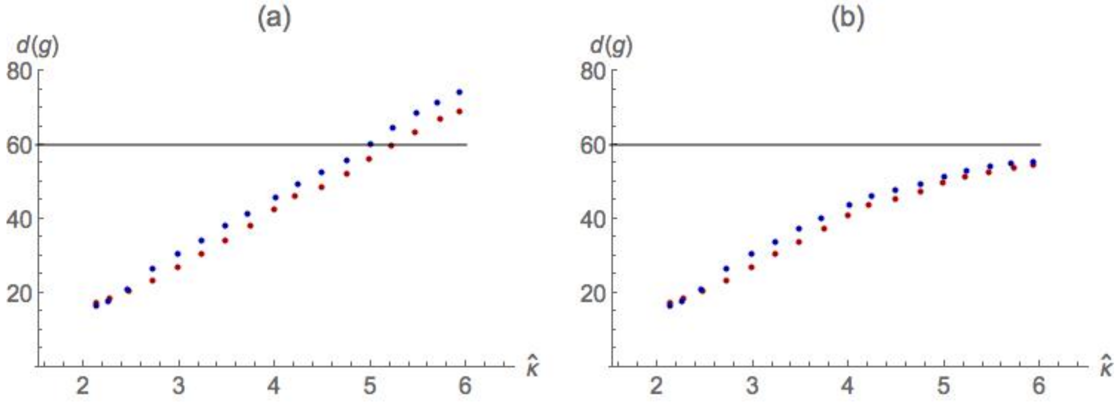

Fig. S1. The relationship between simulation fertility parameter, κ, and average degree number of relatives, $d(g)$. Panel (a): before truncation at 60; panel (b) after truncation. X-axis: the actual fertility in the population, i.e., measured after the standardisation of the population size to n=500 or n=2000; y-axis: number of relatives in the same generation (siblings or first degree cousins). Red: n=500, blue: n=2000. Each dot represents the average of the bin of the size 0.5 in terms of κ. The black horizontal line represents the truncation threshold.

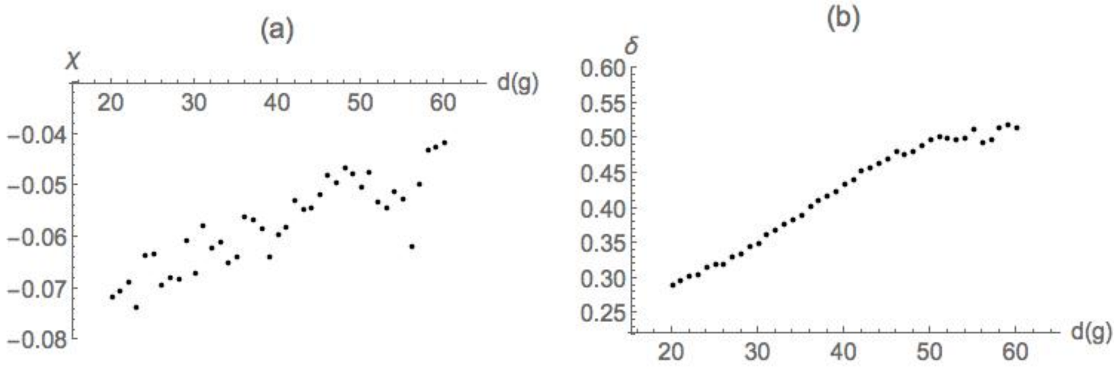

Fig. S2. Differentials of Fig.1 based on group size. Panel (a, b) x-axis: the number of relatives out of the maximum possible 60. Panel (a) y-axis: clustering coefficient differential: χ_{2000}-χ_{500}. Panel (b) y-axis: average graph distance differential: δ_{2000}-δ_{500}.
